# Night skies through animals’ eyes—Quantifying night-time visual scenes and light pollution as viewed by animals

**DOI:** 10.3389/fncel.2022.984282

**Published:** 2022-10-06

**Authors:** Anna Lisa Stöckl, James Jonathan Foster

**Affiliations:** ^1^Department of Biology, University of Konstanz, Konstanz, Germany; ^2^Centre for the Advanced Study of Collective Behaviour, University of Konstanz, Konstanz, Germany; ^3^Zukunftskolleg, Universität Konstanz, Konstanz, Germany

**Keywords:** natural visual scenes, all-sky imaging, dim light vision, light pollution, ALAN, hawkmoth, mouse, fruitfly

## Abstract

A large proportion of animal species enjoy the benefits of being active at night, and have evolved the corresponding optical and neural adaptations to cope with the challenges of low light intensities. However, over the past century electric lighting has introduced direct and indirect light pollution into the full range of terrestrial habitats, changing nocturnal animals’ visual worlds dramatically. To understand how these changes affect nocturnal behavior, we here propose an animal-centered analysis method based on environmental imaging. This approach incorporates the sensitivity and acuity limits of individual species, arriving at predictions of photon catch relative to noise thresholds, contrast distributions, and the orientation cues nocturnal species can extract from visual scenes. This analysis relies on just a limited number of visual system parameters known for each species. By accounting for light-adaptation in our analysis, we are able to make more realistic predictions of the information animals can extract from nocturnal visual scenes under different levels of light pollution. With this analysis method, we aim to provide context for the interpretation of behavioral findings, and to allow researchers to generate specific hypotheses for the behavior of nocturnal animals in observed light-polluted scenes.

## Introduction

Many of the world’s animals, from tiny insects to large mammals, are active during the night. These species face a dramatic change in their sensory environment, as the available light varies more than one million-fold between day and night (Johnsen et al., 2006). This critically limits the information available to the sense of vision, which is fundamental to locomotion control, orientation and navigation, and also heavily relied upon for foraging, predator avoidance, mate detection and communication. Yet, colonizing this challenging diel niche comes with great benefits in avoiding both competitors and predators (Wcislo et al., 2004), thus making evolutionary adaptations to the low light conditions worthwhile. Over millions of years, intricate nocturnal ecosystems have thus evolved, with unique predator-prey interactions (Park, 1940), specialized pollinators (Macgregor and Scott-Brown, 2020; Walton et al., 2020) and even further subdivision of the night into temporally distinct niches (Greiner et al., 2007; Narendra et al., 2011; Stöckl A. L. et al., 2017; Tocco et al., 2019). Counterintuitively, these fragile ecological communities, defined by the scarcity of light, are now threatened by what might be considered a benefit for other species that rely on vision: increasing levels of light at night (Longcore and Rich, 2004; Gaston et al., 2012; Falchi et al., 2016). This artificial light at night (ALAN) can occur either as isolated concentrations of light (i.e., street lights), or an increase in the brightness of the entire sky (i.e., through sky glow) (Longcore and Rich, 2004). However, such ALAN is extremely challenging for visual systems that are so well-adapted to using even the last available photon, as they suddenly encounter an overabundance of light in nocturnal visual scenes.

To date, the dominant paradigm in the study of light-pollution’s effects on nocturnal animals has been to observe individual behavioral and physiological effects in an attempt to infer larger-scale network effects, in which light introduced to the environment can be demonstrated to alter the ecosystem as a whole (Longcore and Rich, 2004; Gaston et al., 2012; Grubisic et al., 2018; Giavi et al., 2021; Gaston and de Miguel, 2022). By comparison, the visual mechanisms themselves have received relatively little attention. This provides those working on mitigating ALAN’s damaging effects with evidence to bolster their case, but limited tools with which to explain these effects or, better yet, to make policy proposals–a “what,” but not a “how” or a “why.” In this article, we make the case for combining the ecological approach to the study of light pollution, with the paradigms, methods and tools developed in the fields of visual ecology and dim-light vision outlined below. We first provide an overview of nocturnal visual environments, and the typical forms of light pollution observed, and then go on to describe the visual systems of nocturnal animals and how they are adapted to low light conditions. Based on this, we present estimates of how light pollution alters the visual percepts of different nocturnal animals, based on realistic ocular modeling of light information from natural terrestrial visual scenes.

## Light and visual habitats

**FIGURE 1:**
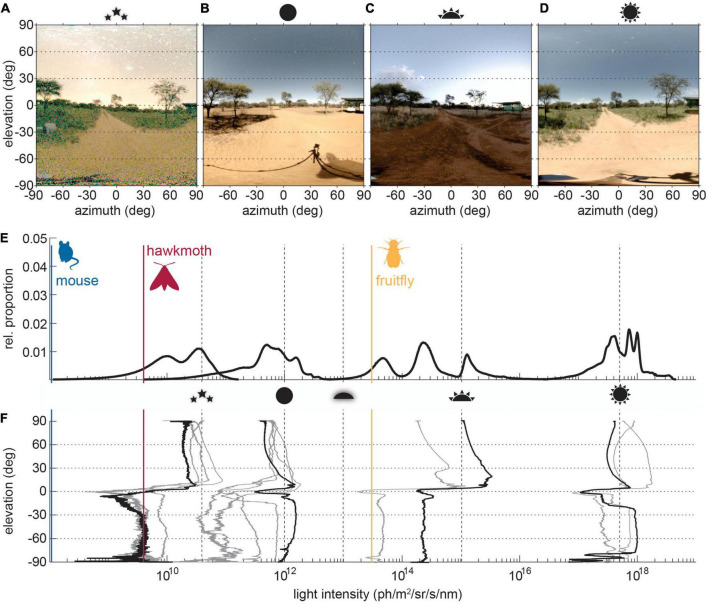
Light intensity distributions in visual scenes across times of day. **(A–D)** The same visual scene near Vryburg (26°23′55.82″ S, 24°19′37.380″E) in South Africa, photographed Southward under different celestial illumination conditions as indicated by the icons (starlight on a moonless night, full moon light with moon above 60° elevation, sunset, noon). **(E)** The relative proportion of pixel intensities and the **(F)** average pixel intensies across scene elevation. Panel **(E)** shows the average brightness histograms for *starlight*, *moonlight*, *sunset/runrise*, and *sun at noon* of a number of individual scenes with elevation intensity profiles depicted in gray in panel **(F)**. The profiles of the example scenes in panels **(A–D)** are highlighted in black. The dashed lines indicate average light intensities under typical celestial conditions (*max. starlight*, *max. moonlight*, *end of twilight*, *sunset/sunrise* and *max. sunlight*). The relative scaling of these were taken from Warrant (2008), while the scale was anchored in absolute terms to the intensity for sunlight from Nilsson and Smolka (2021). The visual thresholds of the three indicated model species [mouse *Mus musculus* (Umino et al., 2008), hawkmoth *Deilephila elenor* (Stöckl et al., 2016a), fruitfly *Drosophila melanogaster* (Currea et al., 2022)] were based on behavioral and physiological measurements of the response thresholds to moving contrast in optmotor-type tasks, both for comparability and because of the high ecological relevance of these tasks for animal behavior.

The vast majority of photons animals observe are not produced here on Earth. For millions of years the visual worlds of animals were lit (with the notable exception of deep-sea environments) by light from the sun; direct, scattered by the atmosphere or reflected by the moon; or from the stars. In the terrestrial world, any earthbound light sources are generally swamped by extra-terrestrial light sources, with exceptions such as lightning flashes or volcanic eruptions being either fleeting, ephemeral, or both. So it is that the visual systems of most animal species evolved to suit the available sunlight (Figures 1C,D), moonlight (Figure 1B), and starlight (Figure 1A), and their typical spectra, intensities, and circadian schedule.

While natural scenes range in brightness across more than six log units (Figure 1E; Johnsen et al., 2006), the range within an individual scene is typically limited to 2 log units (Figure 1F; Nilsson and Smolka, 2021). Around sunset, ambient illumination at visible wavelengths (400–700 nm) is around 10^18^ photons m^−2^s^−1^, dropping to 10^17^ photons m^−2^s^−1^ as the sun reaches the horizon (Johnsen et al., 2006). After sunset, a moonlit night may be as bright as 10^15^ photons m^−2^s^−1^, while a starlit night is around 10^13^ photons m^−2^s^−1^ (Johnsen et al., 2006). When clouds or vegetation then block moonlight and starlight from view, ambient illumination can reach a low of 10^12^ photons m^−2^s^−1^ (Foster et al., 2021). Celestial cycles therefore provide a benchmark for total light available, but these changes need not be uniform across all regions within a scene. Above the horizon, the brightness distribution is dominated by visible celestial bodies (sun, moon, and stars) and blue skylight (solar or lunar) (Nilsson et al., 2022) as well as the dim background of airglow (produced by spontaneous emissions in the ozone layer) visible only on dark nights (Johnsen, 2012). Below the horizon, the observer views mainly reflected light, diffuse reflections from matte objects and specular reflections from shiny ones. On a clear day, the scene may be as bright below the horizon as above it (compare Figures 1B,D and respective profiles), thanks to direct sunlight and moonlight reflected from earthbound surfaces (Nilsson and Smolka, 2021; Nilsson et al., 2022). When the sun and moon are below the horizon, or clouds cover the sky, skylight, starlight, and cloudlight produce diffuse illumination that typically results in a much dimmer scene below the horizon (Figures 1A,C and respective profiles). Subtle changes in spectrum across different solar elevations and between scenes illuminated by the sun, moon and stars can also contribute to changes in diffuse reflections within a scene (Johnsen et al., 2006). It is the detection of these contrasts, between the sky and horizon, objects and their backgrounds, and celestial bodies and empty sky, to which animal vision is adapted. Achieving this, however, requires vastly different functionality within a visual system under brighter and dimmer conditions.

**FIGURE 2:**
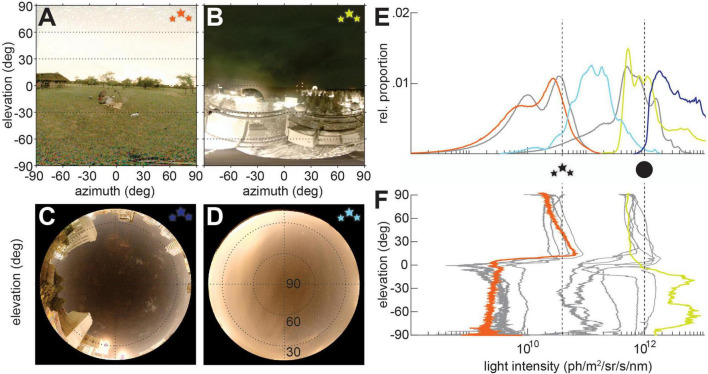
Nocturnal visual scenes with light pollution. **(A–D)** Nocturnal visual scenes on moonless nights with different types of light pollution. **(A)** Starry sky in rural Limpopo (24°43′46″S, 27°57′06″E), South Africa with sky glow visible at the horizon. **(B)** Nocturnal scene in Sodankylä, Finland, showing a weak aurora borealis in the sky, and direct light pollution relfected by the snowy ground below the horizon. **(C)** Sky at the University of the Witwatersrand, South Africa, with direct light pollution from surrounding buildings, and **(D)** sky in Östra Odarslöv, Sweden, showing clouds lit up by sky glow. Lines in panels **(C,D)** indicate 60 and 30° elevation. **(E)** The relative proportion of pixel intensities [colors correspond to those of the star icons for scenes depicted in panels **(A–D)**, with the average intensity profiles for *starlight* and *moonlight* from Figure 1E in gray] and the **(F)** average pixel intensies across scene elevation [the elevation intensity profiles from Figure 1F in gray, and the elevation profiles of the scenes in panels **(A,B)** in color].

The industrial age threw these reliable features of day and night into disarray. Electric lighting introduced an unparalleled source of earthbound light into the environment, both directly, when these lights are in the animal’s field of view (Figures 2A,C), and indirectly, when this stray light projects up into the atmosphere and is returned to earth in the form of skyglow (Figures 2B,D). This ALAN dramatically alters the light environment, raising the ambient light intensity by 1–4 log units (Foster et al., 2021), obscuring the stars from view for human and animal observers alike, and introducing luminous objects into the light field that can outshine the full moon (Figures 2E,F). Increasing awareness of the impacts of human activity on the environment have led to a great interest in the impact of excess light on the structures of ecosystems (Bennie et al., 2015; Grubisic et al., 2018), and number of specific behaviors, such as flight to light (Baker and Sadovy, 1978; Doren et al., 2017; Wakefield et al., 2017), shore migration (Torres et al., 2020), and predation by insectivores on moths attracted to streetlights (Wakefield et al., 2015).

Here we introduce methods that help us understand what information animals can extract from a visual scene when their eyes are adapted to the intensity of ambient illumination.

## Nocturnal vision

Even though the signal amplitude available to the visual system at night is only a minute fraction of that during the day (Figures 1E,F), many animals possess exquisite night vision. The elephant hawkmoth *Deilephila elpenor*, for example, can distinguish flower colors even under starlight intensities (Kelber, 2002), the tropical sweat bee *Megalopta genalis* skillfully approaches and securely lands on its nest stick in the dead of night in the rainforest (Warrant et al., 2004), and the night-migrating indigo buntings are guided by patterns of stars on their journey from North- to Central-America (Emlen, 1967a,b).

These are representative of typical nocturnal animals that invest in specializations of their eyes and nervous systems to make the most of the sparse visual information available. But why evolve such a system, rather than relying on other senses for the same tasks? The answer may be that it is not straightforward to replace the unrivaled depth of information the visual sense provides (Warrant and Johnsen, 2013; Warrant et al., 2020): high spatial- and high-temporal resolution information in several separate wavelength channels, which reaches from distances of only millimeters to many light-years (when viewing the starry sky) (Foster et al., 2018). While some of the information vision provides can be substituted by other senses at night, for example by olfaction for food localization via the odors the food source emits (Healy and Guilford, 1990; Stöckl et al., 2016c), visual information remains crucial for control tasks in relation to remote objects. Such objects, including predators or prey that sparsely emit sound or odors, are almost impossible to localize without vision–except with active sensing (Nelson and MacIver, 2006). And even species with active sensing alternatives, such as bats, use vision for obstacle control (Jones and Moss, 2021). One behavior in particular that cannot be executed with other passive senses, is flight (Davies and Green, 1994). Flying animals need to obtain rapid and remote information about their three-dimensional environment, to detect obstacles and changes in their own body position (Srinivasan et al., 1999; Bhagavatula et al., 2011). Thus, for flying animals in particular, but also for fast moving walkers, negotiating their environment on the search for food, mates or to avoid predators, vision is essential. Thus, many animals exploit even the sparsest light sources to control a range of behavioral tasks, using visual specializations that let them detect intensities down to single photons (Reingruber et al., 2013; Honkanen et al., 2014; Stöckl et al., 2016a; Tinsley et al., 2016; Stöckl A. L. et al., 2017).

A separate reason to rely on vision at night is not despite but because of the low availability of visual signals: glow worms and fireflies generate their own light signals for communication, and thus benefit from the low background levels of light at night, to produce singular and far reaching communication signals. Their visual challenge is similar to that of other nocturnal insects, in particular since the animals need to safely navigate toward their visual beacons (Kahlke and Umbers, 2016).

## What restricts vision at night?

To understand the specializations for vision at night, a closer look at the challenges generated by the low intensities is required. The most fundamental limit of any light detection arises from the physical nature of light. Light particles, or photons, interact in a quantal fashion with the photopigments in the receptors that detect light in animal eyes (Yeandle, 1958; Lillywhite, 1977), or camera sensors (Yadid-Pecht et al., 1997; Tian et al., 2001). However, photons reach any possible surface in a stochastic manner (Rose, 1942; De Vries, 1943), so that the number of photons that arrive within a given area fluctuates over time. This uncertainty in photon arrival–and consequently absorption–is termed photon “shot noise” (Schottky, 1918). It sets the absolute noise limit for any visual reconstruction. Irrespective of how sensitive a light detector is, if the photon shot noise is too high, the original signal received by the detectors cannot be reliably reconstructed. The variance in photon arrival is governed by Poisson statistics (Rose, 1942; De Vries, 1943), so that the signal-to-noise ratio (SNR), the “detection criterion” required to reliably infer the original image, equates to the square root of the signal (N) (Snyder, 1979; Land, 1981):


(1) $$\text{SNR}=N/\sqrt{\text{N}}=\sqrt{\text{N}}$$

Thus, the SNR is much smaller for lower photon catches–as measured at night–than for the high ones during the day. It is important to note that the SNR sets not only the limit for the absolute visual threshold, or absolute visual sensitivity, but also the limit for an animals’ contrast sensitivity: the ability to discriminate between different light levels. Contrast sensitivity forms the basis of spatial vision, for which the inputs of neighboring visual units, representing separate “pixels” of the image, need to be discriminated (Snyder, 1977; Snyder et al., 1977), as well as temporal vision, where the signal at consecutive time points needs to be resolved (Hornstein et al., 1999; Donner, 2021). As light levels decrease, the SNR decreases at higher spatial (Stöckl A. et al., 2017) and temporal frequencies, thus reducing the maximum achievable resolution of the visual system. Thus, the SNR at the photoreceptors and subsequent visual processing sets the limit for absolute visual detection and contrast vision, which is crucial for most visual behaviors.

However, this photon-based description of the SNR applies to a perfect sensor–with no loss in photon signal upon arrival at the eye, and no further sources of noise. Eyes are far from perfect sensors, though. Only a fraction of the photons arriving at the eye are absorbed by photopigments in the receptors (Warrant and Nilsson, 1998). Moreover, the absolute detection and accuracy of the photoreceptors is limited by “false positive” activations of the biochemical phototransduction cascade by thermal energy. The resulting electrical responses are indiscriminable from real photon responses, thus constituting the physiological limit for visual detection (Barlow, 1956; Aho et al., 1988, 1993). This so-called “dark variance” (σ*2D*) is relatively low in insects [ca. 10 false alarms per hour at 25°C in locusts (Lillywhite and Laughlin, 1979)], but is distinctly higher in vertebrates [ca. 360 false alarms per hour at 20°C in toads (Baylor et al., 1980)].

In addition, the photoreceptors’ responses to single photons vary in amplitude, latency and shape because of variations in the transduction cascade (Lillywhite and Laughlin, 1979; Howard et al., 1984). In dim light, this so-called “transducer noise” (σ*2T*) has an equal contribution to the total noise variance as photon shot noise, and at higher light intensities by far exceeds it in insects (Lillywhite and Laughlin, 1979; Laughlin and Lillywhite, 1982; Howard and Snyder, 1983). Thus, noise sources intrinsic to the sensory and nervous system decrease the sparse signal reliability in very dim light further, giving an estimate of the total noise as (Snyder et al., 1977; Cohn, 1983; Warrant, 1999):


(2) $$\text{Noise}=\sqrt{\left(\text{N}+\sigma \,2\,D+\sigma \,2\,T\right)}$$

**FIGURE 3:**
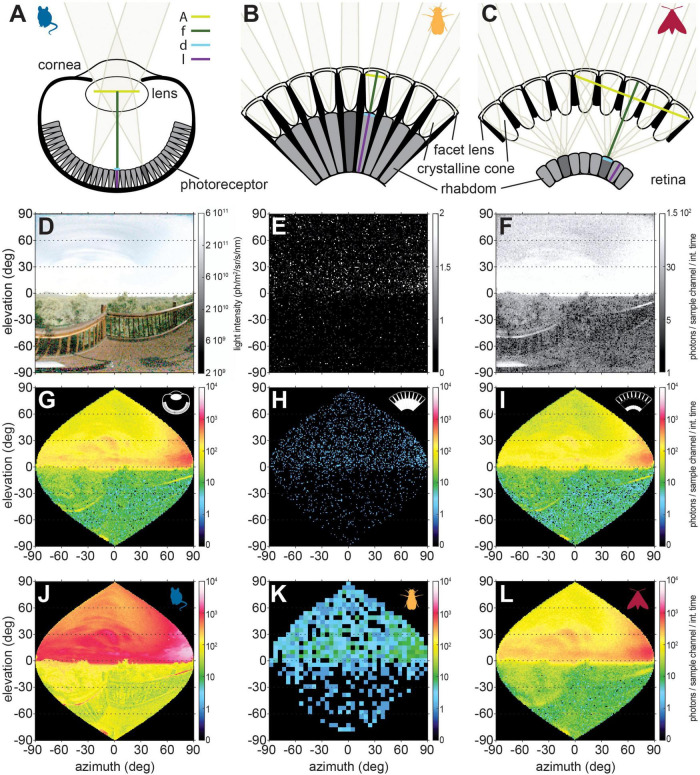
The three most common eye designs in animals. **(A)** In a camera eye, light is focused by the cornea and lens on to the photoreceptors in the retina. This eye design is found in all vertebrates and is also common in the main eyes of molluscs and arachnids. *A*, *f*, *d*, *l* correspond to Eq. 3. **(B)** Apposition compound eyes are made of repeating units, the ommatidia, which are comprised of a facet lens, crystalline cone and the a group of photoreceptors, the retinula. The retinula cells of all ommatidia form the retina. Light is focused by the cornea and crystalline cone of each ommatidium onto the underlying rhabdom, the photosensitive tissue of the retinula cells. In a classical apposition eye, each ommatidium thus constitutes a sampling unit that views a “pixel” of the image. This eye design is commonly found in diurnal insects. **(C)** In superposition compound eyes, light is focused by the corneas and crystalline cones of a large number of ommatidia across the clear zone (cz) onto one rhabdom. Therefore, this eye design is typical for nocturnal insects, which require improvements in relative photon catch. **(D)** Nocturnal scene at moonlight recorded by the camera. Photon count of this scene simulated for **(E)** an apposition, and **(F)** a superposition compound eye [photon count is similar to the camera eye, compare panels **(G,I)**]. All eye types were modeled with photoreceptors of the same width and length, the same spatial resolution (for both the visual angle covered by sampling units, and for the photoreceptor acceptance angle), the same effective apertures for camera and superposition eye, as well as the same facet aperture in both compound eyes (see Table 1 for parameters). The noise levels include photon shot and transducer noise of the same magnitude. **(G–I)** Pixels were resampled to represent the spatial sampling units of a perfectly spherical eye. **(J–L)** The same scene viewed through the eye of a mouse, fruit fly, and elephant hawkmoth (see Table 1 for parameters).

Taken together, at only a few photons per sampling time, the variance of the visual signal is much higher than at higher average photon catches (compare Figures 3E,F). This high uncertainty in the available signal challenges nocturnal vision and requires specializations to improve the low signal to noise ratios in dim light.

**TABLE 1:** Parameters of the different eye types used for photon estimations.

	Mus musculus	Drosophila melanogaster	Deilephila elpenor	Model eye
Facet diameter *D* (μm)	–	16.5	27.6	25
Focal length *f* (μm)	2600^1^	21.4	669	
Spatial sampling base Δφ (°)	0.55^3^	5	1.12	1
Photoreceptor group or rhabdom diameter *d* (μm)	5^3^ × 1.4^2^	7.1	11.3	10
Photoreceptor length l (μm)	23.6^2^	60	165	100
Effective aperture diameter *A*_*e*_ (μm)	2000^1^	33	696	1000
Total number of facets in aperture *n*_*F*_	–	6	577	1111
Acceptance angle Δρ (°)	0.55^3^	8.23	4.05	1
Integration time (ms)	0.324^4^	0.02	0.027	0.050
Spatial summation angle (°)	6.58^5^	6	8.25	–
Number of summed units *n*_*Sum*_	210	6	79	–
Tau	0.65^6^	0.8^7^		0.8
Kappa	0.34^7^	0.5^7^		0.5
K	0.035^7^	0.0067^7^		0.0067
Total noise	$\text{Noise}=\sqrt{\left(\text{N}+\sigma \,2\,T\right)}=\sqrt{\left(2\,N\right)}$			

The modeling parameters were sourced from the following studies: *Mus musculus* (1) (Geng et al., 2011), (2) (Fu and Yau, 2007), (3) calculated from the dendritic field of a rod bipolar cells, given the eye’s focal length (Behrens et al., 2016), (4) (Donner, 2021), (5) calculated from on ON and OFF alpha ganglion cell receptive fields, given the eye’s focal length (Baden et al., 2016), (6) (Henriksson et al., 2010), (7) (Warrant, 1999), *Drosophila melanogaster* (Gonzalez-Bellido et al., 2011), and *Deilephila elpenor* (Stöckl A. L. et al., 2017).

## Anatomical modifications to increase visual sensitivity

To use vision under such adverse conditions, animals need to overcome the challenges that high noise and low signal levels pose to obtaining reliable information for the behavioral tasks at hand–or wing. Improving the SNR can be achieved through directly increasing the number of photons collected by the eye. To understand how eyes can increase sensitivity, it is worth considering the optical sensitivity, a measure for the light capture ability of a visual system. The higher it is, the more light is captured. For extended scenes, this can be expressed as (Land, 1981; Warrant and Nilsson, 1998; Warrant, 1999):


(3) S=π4⁢A2⁢ 1.13⁢(df)2⁢(k⁢l2.3+k⁢l)

In this equation, *A* is the diameter of the aperture, *d* is the photoreceptor diameter, *f* is the focal length of the eye, and *kl*/(2.3 + *kl*) is the fraction of light absorbed, where *k* is the absorption coefficient of the photoreceptor and *l* its length. As Eq. 3 shows, the number of photons each visual unit detects at a given integration time can be maximized by modifications that increase the aperture diameter (*A*) or decrease the focal length (*f*), and that increase the photoreceptors’ diameter (*d*) or length (*l*).

Increasing the cross-section area *d*^2^ of the photoreceptor and its length *l* (or more precisely the volume of tissue presenting photopigment within the receptor) increases the potential photon catch. This is especially important, because the absorption efficiency *k* of every single photopigment is relatively low [ranging between ca. 0.005–0.01 μm^–1^ in arthropods and 0.025–0.05 μm^–1^ in vertebrates (Warrant and Nilsson, 1998)]. Efficient absorption requires stacking membranes with photopigments. The fraction of absorbed white light γ is therefore directly proportional to the length of the photoreceptors *l*: γ∝*kl*/(2.3 + *kl*) (Warrant and Nilsson, 1998).

In addition to optimizing the probability of photon absorption, nocturnal eye designs are optimized to focus a wider cone of light onto the photoreceptors than typical diurnal eyes (Warrant and McIntyre, 1991). This is achieved via a large aperture combined with a shorter focal length. This relationship between aperture and focal length is expressed in an eye’s F-number: *F* = *f*/*A*. In eyes and photographic cameras, low F-numbers denote high sensitivity and vice versa.

### Camera eyes

Nocturnal animals with camera type eyes (Figure 3A), as possessed by all eyed vertebrates, generally have large lenses with wide pupils (the effective aperture of the eye) and shorter focal lengths than their diurnal relatives. For comparison, a dark-adapted human eye has an F-number of 2.1 (Westheimer, 1970), while the F-numbers of nocturnal primates [Lesser Bushbaby: 0.71 (Kirk, 2006)] or nocturnal birds [barn owl: 0.83 (Pettigrew, 1983)] are distinctly lower. Invertebrates also follow this trend: the nocturnal spider *Dinopis subrufus* achieves its remarkable sensitivity with the largest lens of any terrestrial arthropod (Blest and Land, 1977), which together with a short focal length results in the very low F-number of 0.6.

### Apposition compound eyes

Compound eyes, the most common eye type among insects and crustaceans, are composed of many “little eyes” or ommatidia, which constitute the visual units of the eye. In apposition compound eyes (Figure 3B), each ommatidial facet lens focuses light onto the photoreceptors comprising a visual unit, thus acting as the effective aperture of the eye. Because of the small size of individual facets, which constitute the eyes’ aperture, apposition compound eyes are limited in sensitivity (compare the photon distributions in Figures 3G,H) and are mostly found in diurnal arthropods. There are a few notable exceptions including nocturnal mosquitoes (Land, 1997), the tropical halictid bee *M. genalis* (Greiner et al., 2004b; Warrant et al., 2004), and nocturnal ants (Menzi, 1987; Moser et al., 2004; Narendra et al., 2011). As in camera eyes, these nocturnal apposition eyes generally have larger apertures, in their case facets, than their diurnal relatives, but even these modifications do not increase sensitivity to the level of nocturnal camera eyes [i.e., sensitivity of 2.69 in the nocturnal sweat bee (Greiner et al., 2004b)]. Moreover, the increase in sensitivity through larger apertures is achieved at the expense of spatial resolution, since an eye with larger facets can “fit” fewer of them than the same eye with narrower facets, and thus resolve fewer “pixels.” The only way to escape this trade-off is to increase overall eye size to retain a comparable spatial resolution with wider facet apertures (Jander and Jander, 2002; Greiner et al., 2004b).

### Superposition compound eyes

An optical solution to the trade-off between sensitivity and spatial acuity is provided by the most prominent eye type of nocturnal insects: superposition compound eyes (Figure 3C). In this eye type, the pigment that separates the ommatidia in apposition compound eyes can withdraw, which leaves a wide clear zone between the crystalline cones underlying the facet lenses and the retina. The crystalline cones focus light from one point in space through multiple facets onto a single rhabdom (Exner, 1891; Nilsson, 1989). Thus, the effective aperture of this type of eye comprises all facets with a shared optical axis for this point in space. We can therefore extend Eq. 3 by *n_f_*, the number of facets contributing to the superposition aperture, and hence obtain the overall aperture area of the superposition eye as π/4*D*^2^*n*_*f*_ where *D* is the facet diameter.


(3.1) $$S=\frac{\pi }{4}\,D^{2}\,n_{f}\,1.13\,{\left(\frac{d}{f}\right)}^{2}\,\left(\frac{k\,l}{2.3+k\,l}\right)$$

This increased aperture greatly increases the optical sensitivity of the eye, as noticeable by the low F-numbers of the nocturnal elephant hawkmoth at 0.72 (Warrant, 1999) or the dung beetle *Onitis aygulus* at 0.60 (McIntyre and Caveney, 1998). Thus, in superposition compound eyes, as in camera eyes, sensitivity can be increased by increasing the aperture without affecting the spatial sampling base (nominal spatial resolution) of the eye, resulting in comparable visual sensitivities between these types of eyes, given comparable eye features (compare Figures 3G,I). Nevertheless, due to the intricate constraints on their optics, superposition compound eyes are often limited by spherical aberration, which results in a blurring of the image focussed on the retina and thus reduces spatial acuity below the theoretical maximum (Caveney and McIntyre, 1981; McIntyre and Caveney, 1985; Warrant and McIntyre, 1990). This can be seen by comparing Figure 3I, which shows a visual scene through a model superposition eye with 1° spatial resolution, and Figure 3L, which shows this scene through the eye of the elephant hawkmoth *D. elpenor*. This hawkmoth possesses an anatomical sampling base (angular separation of ommatidia or interommatidial angle △φ) of 1.12°, but the effective spatial resolution of the photoreceptors (photoreceptor acceptance angle △ρ) is only 4°, resulting in considerable loss of resolution compared to Figure 3I.

## Modeling photon catches and spatial resolution

As discussed in the previous paragraph, the combination of anatomical eye parameters, including aperture, focal length, photoreceptor diameter and length determines the visual sensitivity, while also setting the limits for spatial resolution.

To calculate the photon catch each sampling channel of an eye receives from a natural visual scene, the visual sensitivity S (Eq. 3) is multiplied with the measured radiance in a given time interval △*t*, generally the channels’ integration time, as well as scaled by the quantum capture efficiency of the transduction cascade κ, and the fraction of incident light transmitted by the optics τ of the eye (Warrant, 1999):


(4) N=π4⁢A2⁢ 1.13⁢(df)2⁢τ⁢κ⁢(k⁢l2.3+k⁢l)⁢△⁢t⁢I

(4.1) N=π4⁢A2⁢ 1.13⁢△⁢ρ2⁢τ⁢κ⁢(k⁢l2.3+k⁢l)⁢△⁢t⁢I

**FIGURE 4:**
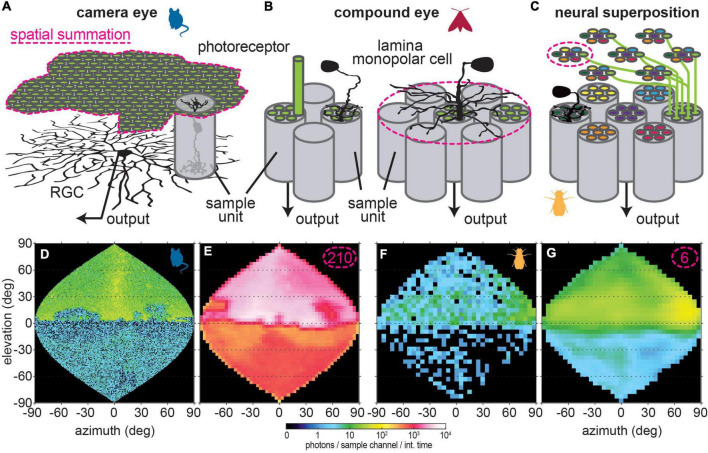
Different types of spatial summation. **(A)** Spatial convergence in the vertebrate retina occurs in two stages: photoreceptors signals are pooled by bipolar cells (*sample unit*), which in turn are integrated by retina ganglion cells (RGC), which transmit visual information to the brain. Note that the tiling of RGCs differs from the of bipolar cells, thus resulting in an altered sampling base after spatial summation. **(B)** In most insects, photoreceptors from one ommatidium of the eye (Figures 2B,C) project as a combined sample unit to a single cartridge in the lamina, where lamina monopolar cells (LMC) integrate their information and relay it to the next brain area. Particularly in nocturnal insects, LMCs with lateral processes that extend into neighboring cartridges can thus pool information across sample units, and perform spatial summation as a result. Note that the tiling of these LMCs corresponds to that of the photoreceptors, so that the spatial sampling base does not change when LMCs sum information in space. **(C)** The neural superposition eyes of Diptera possess a special organization to integrate information spatially: the photoreceptors in each of their ommatidia view different points in space, as represented here by different colors for photoreceptors in each ommatidium. In neighboring ommatidia, one receptor each views the same point in space (same color code). These receptors project to the same cartridge in the lamina, so that the lamina units represent on “pixel” of the image. This way, light representing this “pixel” is focused by six neighboring facets. Neural summation of information across neighboring sample units takes place by integrating information from the photoreceptors in one ommatidium. **(D)** The same starlight scene as in Figure 1A viewed through a mouse eye without and **(E)** with spatial summation. **(F)** Same moonlit scene as in Figure 3A viewed through a fruitfly eye without and **(G)** with spatial summation.

The term (π*d*^2^)/(4*f*^2^) can also be replaced by the solid angular subtense of the sampling channel’s (Gaussian) receptive field 1.13 △ρ^2^. △ρ is the half-width (full-width at half maximum) of the channel’s receptive field. It is often assumed that the sampling base of the eye is a single photoreceptor–as this is the smallest sensory unit that makes up the retinal mosaic. However, functionally, the sampling base of the visual percept does not depend on the resolution of the sensory sampling, but on the number of separate information channels resulting from this mosaic. Take the case of a classic apposition or superposition compound eye (Figures 3B,C): several photoreceptors are grouped together to form the retinula of a single ommatidium, which view the same visual space. The axons of these photoreceptors (to avoid additional complications arising in color channels, we consider only receptors that terminate in the lamina in our model) transmit information to downstream neurons that pool their information indiscriminately (Rivera-Alba et al., 2011; Matsushita et al., 2022). Thus these photoreceptors in a single ommatidium together form one visual sampling channel. The case in the vertebrate retina becomes somewhat more complicated, since information is first pooled from small groups of photoreceptors by bipolar cells, before these in turn are pooled by retinal ganglion cells (RGCs) (Masland, 2012) (Figure 4A). Individual photoreceptors only constitute separate sampling units in rare cases, and even in those cases they are subsequently sampled via bipolar cells, which constitute the functional sampling units. A number of different bipolar (Behrens et al., 2016) and RGC (Baden et al., 2016) types form parallel visual channels with different spatial sampling bases. Since the RGCs ultimately provide the information channel from the eye to the rest of the brain, one might argue that they constitute the functional sampling base of the retina. However, recent work provides strong evidence for non-linear integration of bipolar cell information by RGCs, suggesting that these function as separate sub-units at least to some degree (Zapp et al., 2022). For the mouse example in our study (Figure 3J), we focused only on rod pathways that are active in dim light, which strongly reduces the number of bipolar cells, and assumed these to be the elementary sampling units. Thus, in Eq. 4 we would have to adjust *d* for the number of photoreceptors integrated by one rod bipolar cell [see for example (Behrens et al., 2016)].

As a consequence, when attempting to obtain a realistic impression of an animals’ view of a natural scene, it is vital to consider the functional visual sampling base relevant to the question (and thus the visual pathway) at hand.

## Dynamic adjustments of visual sensitivity

In addition to the anatomical adjustments that improve visual sensitivity at night, the dynamic range of the photoreceptors and subsequent neural processing are often adjusted to the ambient light intensity. This is important, because for any given neuron the dynamic range is much smaller than the range of light intensities experienced between day and night (Walcott, 1975). The contrast range of single visual scenes at a given light intensity, however, is generally constrained to one, maximum two log units (Figure 1F), and thus more closely matches the dynamic ranges of visual neurons (at about two orders of magnitude response variation) (Laughlin S., 1981; Laughlin S. B., 1981; Srinivasan et al., 1982; Laughlin, 1989; van Hateren and van der Schaaf, 1996). By dynamically matching the visual responses to the current contrast range, the visual system can encode scenes over the entire intensity range an animal experiences. This so-called light adaptation takes place both at the level of the eyes’ optics, and at several processing stages of the visual pathway.

### Dynamic adjustments of the optics

In all three eye types, the amount of light entering the photoreceptors can be regulated dynamically. This is important, because photoreceptors have a limited dynamic range (Walcott, 1975). Like an overexposed camera image, allowing too much light into the eye saturates the photoreceptors, thereby severely compromising visual perception. Thus, a pupil restricts the incoming visual signal to a manageable dynamic range. In vertebrate camera eyes, the iris works as a pupil and contracts to restrict the amount of light entering through the lens (Walls, 1942). In arthropod compound eyes, the pupil is composed of migrating pigment granules. In apposition eyes, pigment typically moves radially toward (light adaptation) and away from the rhabdom (dark adaptation) (Ribi, 1978). In the light-adapted state the pigment absorbs stray photons and additionally sharpens spatial acuity (Land and Osorio, 1990). In superposition eyes, pigment moves longitudinally to separate (light adaptation) or combine the light rays (dark adaptation) from neighboring optical units (Warrant and McIntyre, 1996). The opening or closing of the pupil is controlled by light detected within the eye itself (Nilsson et al., 1992).

For all estimates of animal visual-scene perception in this study, we assumed open camera and superposition pupils. This is realistic for hawkmoths, whose pupil opens at twilight intensities (Stöckl et al., 2016a), while in mice the pupil is only fully open for starlight intensities, and closes gradually with increasing light intensity over a range of at least five orders of magnitude (Bushnell et al., 2016). One important aspect concerning ALAN is to what degree pupil dynamics are affected by light pollution, in particular direct light pollution that appears suddenly, such as passing cars, or streetlights animals encounter while on the move. Do these alter the pupil dynamics, and as a result render the animals subsequently less sensitive when the light has passed, while the pupil still requires some time to open again? Careful measurements of the responses of pupils in camera and superposition compound eyes to dynamic ALAN encounters will be required to address these questions.

### Temporal dynamics of photoreceptors and downstream neurons

As depicted in Eq. 4, the photoreceptors’ response times directly scale the absorbed photons during a single processing interval or integration time. Consequently, in most animal species dark-adapted photoreceptors respond more slowly than light-adapted ones (Laughlin and Weckström, 1993; Juusola and Hardie, 2001; Reber et al., 2015; Stöckl et al., 2016a), thus effectively increasing their integration time, the time during which photons are integrated to generate a response. Just as when we increase the exposure time for a camera, more photons are thus collected, and a higher signal to noise ratio is achieved (Snyder, 1977, 1979; Lythgoe, 1979; Dubs, 1981; Warrant, 1999; Laughlin S. B., 1981; Warrant and McIntyre, 1996). Moreover, integration times tend to be longer in the dark-adapted than the light-adapted state (Laughlin and Weckström, 1993; Juusola and Hardie, 2001; Reber et al., 2015; Stöckl et al., 2016a) and also longer in nocturnal than diurnal species (Laughlin and Weckström, 1993; Frederiksen et al., 2008; Stöckl A. L. et al., 2017; Frolov and Ignatova, 2019; Donner, 2021). Extremely long integration times have been measured in nocturnal toads [1.5 s (Donner, 1989)] and in a deep-sea crustacean [160 ms (Moeller and Case, 1995)]. Such long integration times lead to severe blurring of moving objects (such as predators or prey) or the visual surroundings of animals that move themselves, which makes them challenging for flying animals, or animals that need to chase fast moving prey.

Very similar to the dynamic adjustments in photoreceptors, temporal integration can also occur at other stages of visual processing to improve the SNR, albeit not by directly affecting the photon signals, but by summing visual responses correlated in time, while averaging out uncorrelated noise (Laughlin S. B., 1981; Snyder, 1977, 1979; Lythgoe, 1979; Warrant and McIntyre, 1996; Warrant, 1999; Stöckl et al., 2016a).

For this study, we chose not to address the effects of temporal response dynamics in animal visual systems in detail, because to assess these in relation to natural visual scenes, we would need to process time-varying visual inputs. Obtaining such data is considerably more challenging than obtaining still-image radiance maps–for technical reasons regarding both the camera and animal tracking. The imaging methods applied here [and in Hänel et al. (2018), Jechow et al. (2018)] generally require very long exposure times (e.g., darker than full moon: sky 2–3 min, below the horizon 8 min) for sampling the full intensity range using HDR bracketing (Hänel et al., 2018; Foster et al., 2021; Nilsson and Smolka, 2021), so filming a moving sequence in real time is not possible with this method. While it may be feasible to record an image set at regular intervals along the desired route (e.g., Wystrach et al., 2012) at the necessary exposure times, to simulate the changes in visual information as the animals follows this route, there is potential for significant changes to the visual environment during the time interval required that are far greater than those genuinely experienced by an animal following the route in real time. Moreover, to assess the impact on natural movement input, in particular that generated the animal’s own activity (the majority of movement perceived by terrestrial animals), movement sequences ought to realistically simulate the resulting image velocities and rotations experienced by the animals in question. However, published information on the fine-scale trajectories of nocturnal animals, in particular those of insects, are extremely sparse, even more so within natural environments. This is due to the methodological challenges of detecting small animals under low light conditions, outside of highly constrained and controlled lab environments. However, new tracking methods are continuously developed (Vo-Doan and Straw, 2020), including with potential for night-time applications (Haalck et al., 2020; Walter et al., 2021).

### Spatial summation in the visual system

Just as for temporal integration, spatial summation of visual information, that is integration of information from neighboring sampling units (or “pixels” of the image), can increase the SNR. Since most visual signals are correlated in space (resulting from objects viewed by several neighboring sampling units), integrating information at the same point in time from neural elements processing neighboring “pixels” thus sums this correlated signal and averages out uncorrelated noise (Laughlin S. B., 1981; Snyder, 1977, 1979; Lythgoe, 1979; Warrant and McIntyre, 1996; Warrant, 1999). While spatial summation does not directly act on the photon catch, summing the visual signals generated by photons in the neighboring sampling units thus effectively increases the SNR by a virtual increase in photon numbers per summation sampling unit. This increase is calculated by multiplying the photon estimate in Eq. 4 by the effective number of summed sampling units n_sum_. n_sum_ can be obtained from anatomical analyses, or by measuring the (half-width) acceptance angle △ρ_sum_ of the receptive field of the summing neuron and calculating the number of sampling units within it using the anatomical sampling base of the original visual units △φ (Warrant, 1999):


(5) $$n_{s\,u\,m}=1.46\,{\left(\frac{△\,\rho_{s\,u\,m}}{△\,\varphi }\right)}^{2}$$

Unlike the signatures of temporal summation, the signatures of spatial summation manifest in the morphology of neurons: to integrate from neighboring visual units, neurons with extended processes that contact neurons are required, collecting local visual information (Figures 4A,B). In the vertebrate retina, the photoreceptor signals that are conveyed by bipolar cells are, in turn, integrated by RGCs (Figure 4A). While in the high resolution areas of the eye, such as fovea in many primates, only very few photoreceptors connect to a RGC center, in the visual periphery thousands of photoreceptors can converge onto a single ganglion cell (Roska and Werblin, 2001; Chichilnisky, 2005; Helmstaedter et al., 2013). With such convergence, the spatial resolution of the corresponding visual percept decreases (as a function of the number of photoreceptors connected via bipolar cells to one ganglion cell) to the receptive field half-width of the RGCs, while the sensitivity greatly increases with the number of pooled sampling units (compare Figures 4D,E). Since the RGCs with large receptive fields tile the retina at lower density than the bipolar cells they integrate, this means a conversion to the sampling base of the integrating neurons (compare number of pixels in Figures 4D,E) not only for the spatial resolution, but also for the spatial sampling base of the image.

In non-dipteran insects, spatial summation occurs in the lamina (Stöckl et al., 2020), the first visual processing layer of the insect brain. This neuropil’s main relay neurons, lamina monopolar cells, receive information directly from photoreceptors, relaying it further downstream (Figure 4B). In nocturnal insects, the extent of the lateral processes can be extensive, and reach dozens of neighboring visual units (Strausfeld and Blest, 1970; Ohly, 1975; Ribi, 1977; Wolburg-Buchholz, 1979; Greiner et al., 2004a; Stöckl et al., 2016b). The lamina is divided into retinotopic cartridges, so that each cartridge receives information from one ommatidium, and possesses a set of spatially summing neurons. As a result, the sampling base does not change with spatial summation. Even though the spatial resolution of the visual percept changes, due to the lateral integration of visual information by lamina neurons.

Flies possess an exceptional ocular and laminar anatomical organization, that inherently incorporates spatial summation, the so-called neural superposition compound eye. The photoreceptors of each retinal unit that underly a facet of the compound eye receive light from different neighboring locations in a visual scene (Braitenberg, 1967; Kirschfeld, 1967). All photoreceptors that view the same location, originating in different sampling units of the eye, project to the same unit in the lamina (Figure 4C). Thus, the photoreceptors contributing to viewing one “pixel” of the image receive light through six neighboring facets, as they are located in six neighboring ommatidia. Spatial summation, that is integration of information over neighboring points in space, is achieved by pooling information from the photoreceptors of each ommatidium–since these view different points in space. Practically, this is accomplished by electrically coupling photoreceptors within one ommatidium (Dubs et al., 1981; Dubs, 1982). Because each ommatidium in the fly eye contains six photoreceptors that project to the lamina sample neighboring points in space (Braitenberg, 1967; Kirschfeld, 1967), spatial summation by this neural superposition mechanism is limited in extent–yet it is still effective to make the visual percept more reliable at the limits of the fly eye’s visual sensitivity (Figures 3K, 4F,G).

### Light-dependent dynamics of spatial processing

In both vertebrates and insects, spatial processing in the visual periphery is adjusted according to the ambient light intensity, integrating information in space in dim light and thereby sacrificing spatial resolution to gain sensitivity (van Hateren, 1992; Stöckl A. et al., 2017; Wienbar and Schwartz, 2018). In vertebrates, RGCs linearly integrate the responses of bipolar cells in their receptive fields at low light intensities, while they integrate thresholded individual responses, thus resolving smaller spatial patterns, at higher light intensities (Farrow et al., 2013; Grimes et al., 2014; Mazade and Eggers, 2016). Although the light intensity at which this change occurs has been reported to be at the threshold where vision switches from relying on the less sensitive cone cells to the more sensitive rod cells, the exact luminance can differ for individual ganglion cells, as well as between ganglion cell types, and the ultimate effect on the spatial properties can also differ between RGC types (Tikidji-Hamburyan et al., 2015). In insects, under dim conditions visual information is spatially integrated by lamina monopolar cells, the first visual processing stage downstream from the photoreceptors, (van Hateren, 1992; Stöckl et al., 2020). In bright light, their receptive fields narrow to a single visual sampling base, and often show evidence of lateral inhibition, similar to the receptive fields of many vertebrate RGCs.

While these light-dependent changes in spatial processing are well described under constant illumination conditions, it is not clear how rapidly this dynamic spatial tuning can switch: experiments in both vertebrates and invertebrates were conducted with a minimum of 5 min light adaptation to a background intensity. However, animals moving within natural light environments, particularly those that include artificial light, experience rapid changes in local and average illumination that can occur within seconds–as animals turn toward or away from light sources. To understand how their visual systems adjust to these changes, further investigations into the speed of these light-dependent dynamics of spatial and temporal tuning are required.

Since visual scenes can greatly differ in local light intensity (Figures 1, 2), both the temporal dynamics of the luminance-dependent processing, and, crucially, their spatial dynamics, are relevant for the visual percept. Studies of motion adaptation in wide-field neurons in insects (Li et al., 2021), and contrast adaptation in vertebrate RGCs found that both local and global processes play a role (Garvert and Gollisch, 2013). In vertebrate RGCs, local and global adaptation contribute with different proportions for different RGC types (Khani and Gollisch, 2017). The stimulus protocol used in Farrow et al. (2013) suggests that the light-dependent changes in spatial processing are also triggered by illumination local to the RGC, rather than the average background intensity. On the other hand, effects of neural modulation on light-dependent adaptations have been described that ranged well beyond the receptive fields of individual ganglion cells (Eggers and Lukasiewicz, 2010). These also feature very prominently in the circadian system (Ribelayga et al., 2008; Allen et al., 2014). Since the circadian system modulates light adaptation in insect eyes (Nilsson et al., 1992), and prominent circadian changes have been observed in the insect lamina (Meinertzhagen and Pyza, 1996), where dynamic spatial processing takes place, it is very possible that dynamic spatial tuning in insects is also modulated by the circadian system. It would therefore be highly informative to determine how local fluctuations in light intensity (such as a street-light in a nocturnal visual scene) alter luminance-dependent spatial and temporal tuning in individual RGCs in vertebrates, and individual lamina neurons in insects–to understand how such spatially dynamic light environments affects the visual percept of nocturnal animals.

## Impact of artificial light at night on an animal’s visual percept

To estimate how visual responses play out for natural scenes, it is crucial to understand both light stimulation and the internal states that cause changes in the spatial (and temporal tuning of visual neurons), as well as the dynamics with which these changes occur. This is all the more important for light polluted scenes, since, almost by definition, these have a broader dynamic range than natural scenes.

### Real-world environments

A noticeable feature of night-time scenes with light pollution is that both direct and indirect light pollution enhance across-scene extremes in light intensity. Skyglow (Figures 2A,D), particularly at the horizon, increases the brightness of the upper half of the scene (Figures 2E,F), while illuminating the ground diffusely, unlike direct illumination from the sun or moon (compare Figures 1E,F). This results in an average intensity-difference between above and below the horizon of more than tenfold. Similarly, direct light pollution on the ground (Figure 2B) greatly increases the brightness in the lower half of the visual field, leaving the sky distinctly dimmer (N.B. the Aurora borealis in this scene alone raised the sky brightness by two orders of magnitude compared to clear starlight). Even though one might assume that the added light could benefit the visual systems of nocturnal animals, which are brought to their limits precisely because not enough light is available (Figures 3E,F, 4D,F), the distribution of the artificial night, both spatially and temporally, can lead to detrimental effects for animal vision.

**FIGURE 5:**
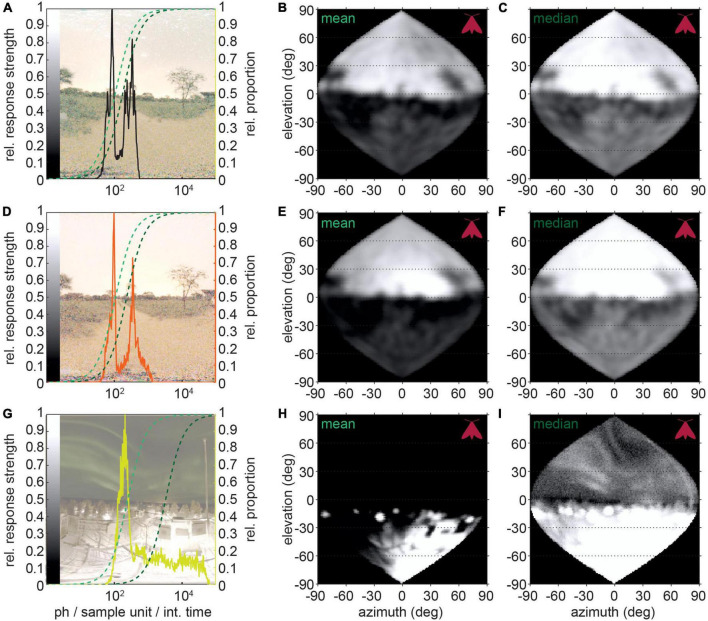
Modeled neural responses to visual scenes with and without light pollution. **(A,D,G)** Natural scenes on moonless nights with different levels of light pollution: **(A)** no light pollution, **(B)** same location with indirect light pollution (sky glow) and **(C)** direct lighting of a high albedo ground. Overlaid are the distributions of photon numbers per sample unit with spatial summation applied for the hawkmoth *D. elpenor*. The photon distributions were mapped to a sigmoidal response curve spanning 2.5 log_10_-units of brightness, which were anchored either to the mean (dark green) or median (light green) of the distribution. Panels **(B,E,H)** show the responses for *mean* mapping, and panels **(C,F,I)** for *median* mapping, resampled to the sampling base of the animal.

This is because, as discussed earlier, visual neurons only have limited dynamic ranges. If the brightness distribution of a visual scene spans many orders of magnitude, the instantaneous mapping to the dynamic range of a neuron results in poor resolution of potentially crucial differences in intensity. For our modeling, we assumed a typical neural response mapping onto two orders of magnitude (corresponding to 95% of the model neuron’s response range) of light intensity (Laughlin S. B., 1981; Srinivasan et al., 1982; Laughlin, 1989; Figures 5A,D). Of the different options for anchoring the mapping reference points we chose to map the center of the response range (inflection point of the response curve) to the *mean* intensity of the scene (Figures 5B,E,H), and to the brightness range that occurs most frequently [*median*, (Figures 5C,F,I)].

We applied this mapping to different visual scenes all viewed through the eyes of *D. elpenor* for comparability. Even though a non-polluted starlit scene (Figure 5A, same as Figure 1A) also has large brightness differences between the sky and the ground, these still map well onto the 2-log-unit range of the neuronal response, so that both contrasts within the sky and within the below-horizon environment are strong. As, a result, if the animal moved its gaze across the scene, the neuron would be instantaneously capable of encoding all the brightness differences, without losing information to saturation or noise at the brightness distribution’s upper and lower ends, respectively. While the ground was of similar brightness in a comparable environment with indirect light pollution (sky glow on the horizon), the distribution was skewed toward higher light intensities (Figure 5D). Even though this effect was quite subtle, it ultimately resulted in the compression of either the dimmer (*mean* mapping, Figure 5E) or brighter (*median* mapping, Figure 5F) intensities, and thus, in a neuron adapted to the average (mean or median) intensity of the scene, a loss of contrast discriminability either above or below the horizon. An extreme version of this unnaturally skewed brightness histogram is created by light pollution combined with a high ground albedo (Figure 5G). Thus, in visual scenes where light pollution generates extreme brightness differences, the neurons’ dynamic range might not be sufficient to resolve contrasts across the entire scene.

Our assumption that neurons adapt only to the average scene brightness is certainly a simplification, since visual neurons also adapt to the local luminance level in combination with global adaptation effects (see *Light-dependent dynamics of spatial processing*). Information about the balance of these two effects in specific neuron types would be crucial to more accurately predict a visual system’s responses to nocturnal light polluted scenes. Likewise, the speed at which these light-dependent adjustments occur would be needed to describe responses to dynamic scenes and shifting gaze. The latter aspect is important, given that similar effects of overextending the dynamic ranges of visual neurons can occur across time, if the light levels the animals experience change rapidly–for example because the light source moves (such as the headlights of a car), or because the animal moves (for example flies past a row of streetlights). While the light intensity animals experience over time also fluctuates with them moving in their environment, these fluctuations mostly remain within the 2.5 log_10_-unit dynamic range of visual neurons (van Hateren and van der Schaaf, 1996). However, flood lights or car headlights viewed on a starry night can exceed this range distinctly (for some examples see Figure 2; Foster et al., 2021), and thus cause similar temporal contrast coding problems as discussed for the spatial domain (Figure 5).

Moreover, in addition to light-dependent neural processing to the sensitivity of visual neurons, it is important to account for changes to their spatial and temporal tuning. Where visual neuron adapt locally to vastly different intensities in different parts of the visual field, how does the visual system form a consistent percept from units that vary locally in both their spatial and temporal resolution–and how does that affect the animal’s behavior? Similarly, if there are rapid changes in spatial or temporal tuning that correspond to rapid changes in light intensity, how does that affect animals’ visually guided behaviors, such as flight control or the detection of foraging targets? If the adjustments occur on longer time-scales than the fluctuation in light intensity, the visual responses would not be optimally adjusted to the current visual environment. For example, an animal might be exposed to bright light sources, resulting in a more constricted pupil and a low level of spatial summation. If it then flies away from the light source, its visual sensitivity would not be sufficient to resolve an unpolluted visual scene (i.e., compare Figures 4D,E,F,G). How impactful these effects would be, would depend on the dynamics of these optical and neural adjustments. For example, in terms of pupil dynamics, there are distinct differences between vertebrates and insects: while a mouse pupil can change its aperture over the course of seconds (Grozdanic et al., 2003), the pupil of a hawkmoth takes an order of magnitude longer to close and more than 10 min to reach its fully open state (Nilsson et al., 1992; Stöckl et al., 2016a).

Thus, to fully understand the impact of ALAN on the visual system of nocturnal animals, we require a better understanding of the spatial response dynamics of visual neurons across a visual scene, as well as the temporal dynamics of dim light-dependent adjustments of spatial and temporal tuning and contrast sensitivity.

## Outlook

As we have seen, ALAN, both direct and indirect, can increase the range of intensities across a visual scene drastically, making it challenging to match this wide range of inputs to the limited dynamic ranges of photoreceptors and downstream neurons (Figure 5). However, often the bright and dim areas cluster in particular regions of the visual field (sky glow in the dorsal hemisphere, direct light pollution often in ventral hemisphere for flying animals). Thus, if animals maintain a stable eye orientation with respect to the horizon, local adaptation (neurons adapting to the regional rather than overall brightness levels) could resolve this dynamic range problem. Since many visual pathways show both local and global adaptation, as well as global state-dependent control, we need to understand how these processes interact to predict how nocturnal animals view scenes with great differences in spatial and temporal intensity.

Artificial illumination not only introduces more light to an animal’s visual environment, but also alters its spectrum at the wavelengths most prevalent in the artificial light source. By increasing the relative intensity in specific regions of the illumination spectrum, this can alter chromatic contrasts between objects and background, which is of particular importance for camouflaged animals (Briolat et al., 2021). Images recorded with RGB cameras, such as those presented in this study, have somewhat limited spectral resolution, but can be used to coarsely estimate chromatic contrast in the human-visible wavelengths, and even into the UV with the correct choice of lens and filters (Troscianko and Stevens, 2015). Recently developed hyperspectral imaging systems (Nevala and Baden, 2019; Tedore and Nilsson, 2019) may make it possible to accurately estimate these changes in chromatic contrast accurately for a range of species with well-studied color vision.

While we focused in this overview on spatial information in images and their representation through animal eyes, temporal changes in the perceived light intensity are of crucial importance for nocturnal animals. Just as they vary over the course of the day, intensities perceived by the eye can also routinely fluctuate by a factor of 100 over shorter time periods, when flying under foliage or in other shaded spaces (Warrant, 2008). These extremes are further enhanced by ALAN, in particular by direct light sources that are many magnitudes brighter than the surrounding natural scenery (compare Figures 1, 2). Emerging from a forest or region of the undergrowth, or turning toward one of these sources after facing a less polluted area, can suddenly change the perceived light intensity on the retina drastically–though how drastically is yet to be reported. How the dynamic adjustments of visual sensitivity, both in the eye (pupil) and the subsequent neural processing (temporal summation, spatial summation), respond to these sudden changes in light intensity, also remains unstudied. While obtaining realistic videos of how a flying insect might perceive a nocturnal visual scene remains technically challenging, studying the dynamic properties of nocturnal visual processing with realistic intensity fluctuations [as has been done for daylight scenes (van Hateren and van der Schaaf, 1996)] is very achievable and would be a crucial first step toward understanding better how sudden changes in light perception as can be caused by direct ALAN affect the visual systems of nocturnal animals.

As described, animals generally do not view static visual scenes, because they themselves are in motion and thus perceive the relative motion of their environment. This also means that, to some degree, they shape the scenes they perceive–they “choose” how long to gaze at certain aspects of a scene, at which distance to pass a street lamp, how fast to walk or fly, and thus shape both the spatial and temporal frequency content, as well as the intensity their eyes perceive. Whether and how nocturnal animals adapt their movements to optimally sample their environment when light conditions change–from day to night, from clear to clouded moon, from a light polluted to unpolluted scene, remains an open and an extremely important question. By actively shaping the visual content they perceive, nocturnal animals might be able to adjust to much more drastic light environments than we might expect–but on the other hand this factor may also make their visual tasks more challenging, if certain movements are required (for example sideways casting for smelling a trail) that deteriorate the visual input (where steady slow forward flying would have been better). As methods for free range tracking improve, we will gain a clearer understanding of the entire cascade of visual perception of natural scenes, from active selection to photon transduction, to subsequent processing. This will greatly enhance our understanding of how animals perceive their night time environments, and where ALAN poses challenges to their visual systems. The methods outlined here can help with this impression–and will be even more powerful once combined with realistic temporal images from animal movement tracks.

## Modeling methods

To obtain the eyes’ spatial resolution, equirectangular images (Figure 3D) were filtered with a Gaussian filter with the half-width set to the acceptance angle Δρ of the visual units (photoreceptors for compound eyes and rod bipolar cells for the mouse). Importantly, the Gaussian filter accounted for the variation in angular distance between pixels along the elevation of the equirectangular images (for details see, Nilsson and Smolka, 2021).^1^ To calculate the photons obtained in each sampling channel per integration time, the light intensity of the imaged scenes was then calculated using Eq. 4, and multiplied by 100 nm to account for the width of a single photoreceptor spectral class (for details see, Stöckl A. et al., 2017; Stöckl A. L. et al., 2017). Spatial summation was implemented by Gaussian filtering with the spatial summation angle, while multiplying the photons per sampling channel by the number of summed units *n*_*S*_. Subsampling the visual scene to the animals’ sampling base was performed by selecting pixels from the equirectangular images at distances equal to the spatial sampling base Δφ (for compound eyes) or the spatial summation angle [for mouse spatial summation, where the sampling base changes from rod bipolar cells to RGCs (see Figure 4A)]. The number of sampling units per elevation was calculated using a sinusoidal projection (for details see, Nilsson and Smolka, 2021).

## Data availability statement

The source data of the analysed images (with calibrated radiance per pixel) is available via Figshare: https://doi.org/10.6084/m9.figshare.20188157.

## Author contributions

AS and JF: conceptualization, methodology, validation, formal analysis, resources, writing – original draft, review and editing, visualization, and funding acquisition. JF: data curation. Both authors contributed to the article and approved the submitted version.

## Footnotes

1
https://github.com/sciencedjinn/elf
